# Facilitatory effect of insulin treatment on hepatocellular carcinoma development in diabetes

**DOI:** 10.1186/s13104-017-2783-6

**Published:** 2017-09-13

**Authors:** Hayato Baba, Makoto Kurano, Takeshi Nishida, Hideki Hatta, Ryoji Hokao, Koichi Tsuneyama

**Affiliations:** 1 Department of Pathology and Laboratory Medicine, Institute of Biomedical Sciences, Tokushima University Graduate School, 3-18-15 Kuramoto, Tokushima, Tokushima 770-8503 Japan; 2 Department of Surgery and Science, Graduate School of Medicine and Pharmaceutical Sciences, University of Toyama, 2630 Sugitani, Toyama, Toyama 930-0194 Japan; 3 Department of Clinical Laboratory Medicine, The University of Tokyo, Tokyo, Japan; 4 Department of Diagnostic Pathology, Graduate School of Medicine and Pharmaceutical Sciences, University of Toyama, 2630 Sugitani, Toyama, Toyama 930-0194 Japan; 5 Institute for Animal Reproduction, 1103 Fukaya, Kasumigaura, Ibaraki 300-0134 Japan

**Keywords:** Diabetes mellitus, Hepatocellular carcinoma, Insulin, Hyperinsulinemia, Hyperglycemia

## Abstract

**Background:**

To evaluate the effect of insulin treatment on the incidence and/or severity of hepatocellular carcinoma (HCC) in a mouse model of HCC based on diabetes.

**Methods:**

We recently reported that neonatal streptozotocin (STZ) treatment causes type 1 diabetes and subsequent HCC in ddY, Institute for Animal Reproduction (DIAR) mice. Newborn male DIAR mice were divided into three groups based on STZ and insulin (INS) treatment. STZ was subcutaneously injected (60 mg/g) into the STZ-treated group (DIAR-nSTZ mice, N = 13) and the STZ/insulin-treated group (DIAR-nSTZ/INS mice, N = 20). A physiologic solution was injected into the control group (DIAR-control mice, N = 8) 1.5 days after birth. Insulin was subcutaneously injected into the DIAR-nSTZ/INS mice according to the following protocol: 2 IU/day at 4–5 weeks of age, 3 IU/day at 5–7 weeks of age, and 4 IU/day at 7–12 weeks of age. All mice were fed a normal diet and were subjected to physiological and histopathological assessments at 12 weeks of age.

**Results:**

DIAR-nSTZ mice had significantly lower body weight and higher blood glucose levels than DIAR-control mice, whereas no significant differences were observed between DIAR-nSTZ/INS mice and control mice. At 12 weeks of age, lower weight of paratesticular fat and higher levels of total cholesterol, triglyceride, and free fatty acids were observed in DIAR-nSTZ mice compared to DIAR-control mice, whereas there were no significant differences between DIAR-nSTZ/INS mice and DIAR-control mice. In the livers of DIAR-nSTZ mice, HCC was observed in 15% of cases, and dysplastic nodules were observed in 77% of cases. In the livers of DIAR-nSTZ/INS mice, HCC was observed in 39% of cases and dysplastic nodules were observed in 61% of cases (*p* = 0.011). Moreover, the average tumor size was significantly larger in STZ/INS-treated mice than in STZ-treated mice. Immunohistochemical analysis demonstrated that the expression of ERK1/2, downstream substrates of insulin signaling that activate cell proliferation, was significantly higher in STZ/INS-treated mice compared to STZ-treated mice.

**Conclusions:**

Insulin treatment promoted, rather than inhibited, the progression of liver carcinogenesis in DIAR-nSTZ mice. Hyperinsulinemia rather than hyperglycemia can accelerate the progression of HCC via insulin signaling.

## Background

Diabetes mellitus (DM) has recently become a suspected risk factor for several malignancies, including cancers of the breast, endometrium, pancreas, and liver [1–4]. The correlation between DM and HCC is particularly strong; a systematic review and meta-analysis of 26 studies conducted in 2005 showed that individuals with DM had a 2.5-fold greater risk of HCC than that in controls [4].

In general, hyperglycemia is considered to increase the risk of developing cancer [5]. A large European cohort study found that blood glucose was a significant risk factor for HCC even after adjustment for other metabolic risk factors [6]. In addition, hyperglycemia is reported to be a significant prognostic factor for HCC [7]. Hyperinsulinemia has also been identified as a strong and independent risk factor for the development of various tumors including breast, colorectal, and pancreatic cancers [8–10]. Accumulating evidence suggests a causative link between hyperinsulinemia and HCC development and progression [11]. Considering these findings, whether or not insulin treatment is appropriate for HCC patients with diabetes needs to be examined. Although such a question remains unanswered, insulin treatment is often administered for HCC patients with DM.

To evaluate the effect of insulin control of blood glucose on the progression of HCC, it is essential to conduct studies in vivo. However, there have been few suitable animal models of diabetes-based HCC. We recently reported that neonatal streptozotocin (STZ) treatment causes type 1 diabetes and subsequent HCC in ddY, Institute for Animal Reproduction (DIAR) mice [12]. In this DIAR-nSTZ model, dysplastic nodules were observed at 8 weeks of age and progressed to HCC by 12–19 weeks of age. Because of rapid neoplastic progression based on diabetes in the DIAR-nSTZ model, this model is considered a promising tool for elucidating a direct link between DM and HCC.

In the present study, the effect of insulin treatment on HCC progression was examined by evaluating the incidence and/or severity of HCC in the murine DIAR-nSTZ model with or without insulin treatment.

## Methods

### Animal models

This study was performed at the Institute of Animal Reproduction in accordance with the criteria outlined in the “Guide for the Care and Use of Laboratory Animals” prepared by the National Academy of Sciences and published by the National Institutes of Health (NIH publication 86-23 revised 1985).

The DIAR (ddy, Institute for Animal Reproduction) mouse strain was established in Japan by inbreeding of the outbred ddy strain. The body weight and visceral fat weight of male and female DIAR mice are slightly higher than that of the ddy mice until 3 months of age and no symptoms of diabetes are observed.

Streptozotocin (STZ) is a beta-cell toxin that induces insulin deficiency and hyperglycemia typical of type 1 DM when given in low doses to mice [13]. In the current study, mice were divided into three groups based on STZ and insulin treatment. At 1.5 days after birth, STZ was injected subcutaneously (60 mg/g) into the STZ-treated group (DIAR-nSTZ mice, N = 13) and the STZ/insulin-treated group (DIAR-nSTZ/INS mice, N = 20), whereas a physiologic solution was injected into the control group (DIAR-control mice, N = 8). The mice were housed based on the treatment group in TPX cages (Okazaki Sangyo Co., Tokyo, Japan) in a non-barrier-sustained animal room maintained at 23 ± 2 °C with 50 ± 10% relative humidity and a 12-h light/dark cycle. All mice were maintained on a normal diet (MF; Oriental Yeast Co. Ltd., Tokyo, Japan) and chlorinated water ad libitum. Insulin (Humalin^®^ N; Eli Lilly and Co., Indianapolis, IN, USA) was injected subcutaneously into DIAR-nSTZ/INS mice using the following protocol: 2 IU/day at 4–5 weeks of age, 3 IU/day at 5–7 weeks of age, and 4 IU/day at 7–12 weeks of age. The mice in each group were sacrificed and examined at 12 weeks of age.

### Histological analysis

Liver samples were fixed in 10% formalin solution, embedded in paraffin, cut into 5-μm thick sections, and then deparaffinized for histological analysis. Hematoxylin and eosin staining, and azan staining for fibrosis were performed using standard protocols.

Immunohistochemical analysis was performed to investigate ERK1 and ERK2 expression. ERK1/2 are downstream substrates of insulin signaling that activate cell proliferation [14]. An anti-ERK1 + ERK2 antibody (rabbit polyclonal; Abcam, Cambridge, UK) was used for immunohistochemistry. After deparaffinization, the specimens were heated with antigen retrieval solution, followed by endogenous peroxidase blocking using 5% H_2_O_2_ in methanol for 5 min. Non-specific binding was blocked by incubation in 5% bovine serum albumin (Sigma-Aldrich, Tokyo, Japan) and the specimens were incubated overnight at 4 °C in pre-diluted primary antibodies. The immunoreaction was visualized using diaminobenzidine (DAB)-chromogen/EnVision™ polymer-horseradish peroxidase (K4001; Dako, Glostrup, Denmark and SK4100; Vector Labs, Burlingame, CA, USA). Sections were lightly counterstained with hematoxylin. A previous study has reported that ERK1/2 translocate to the nucleus from the cytoplasm following activation by upstream substrates of insulin signaling. Therefore, we focused on the nuclear expression of ERK 1/2 [15]. Staining intensity was scored on a three-point scale (−, ±, +): −, nuclear staining seen in 0–20% of tumor cells; ±, nuclear staining seen in 20–50% of tumor cells; and +, nuclear staining seen in more than 50% of tumor cells.

### Statistical analysis

Statistical analyses were performed using the SPSS Statistics version 19.0 software (SPSS Inc., Chicago, IL, USA). The Mann–Whitney U test was used to evaluate statistical differences in weight, laboratory data, and tumor size between the groups. Pearson’s Chi square test was performed to compare the pathological findings and scores obtained from immunohistochemical analysis. Differences were considered statistically significant at *p* value <0.05.

## Results

**Fig. 1:**
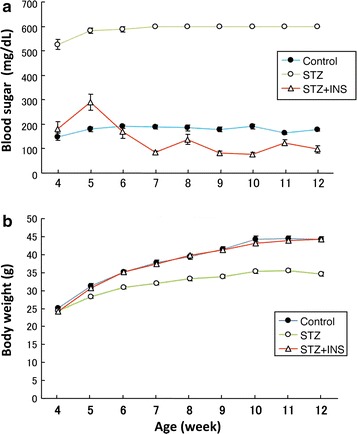
Changes in blood glucose levels (**a**) and body weight (**b**)

**Fig. 2:**
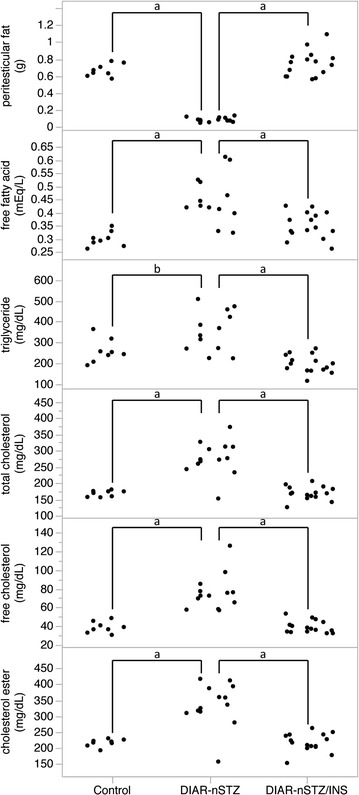
Laboratory data at 12 weeks of age (^a^ *p* < 0.01, ^b^ *P* < 0.05)

The DIAR-nSTZ mice had significantly higher blood glucose levels than the DIAR-control mice but the hyperglycemia improved significantly by insulin treatment (Fig. 1a). In the DIAR-nSTZ mice, body weight loss due to continuous hyperglycemic state was observed whereas no significant difference in body weight was observed between the DIAR-nSTZ/INS mice and the control mice (Fig. 1b). At 12 weeks of age, significantly lower paratesticular fat weight and higher total cholesterol, triglyceride, and free fatty acid levels were recognized in the DIAR-nSTZ mice compared to the DIAR-control mice, whereas there were no significant differences between the DIAR-nSTZ/INS mice and the DIAR-control mice (Fig. 2).

**Fig. 3:**
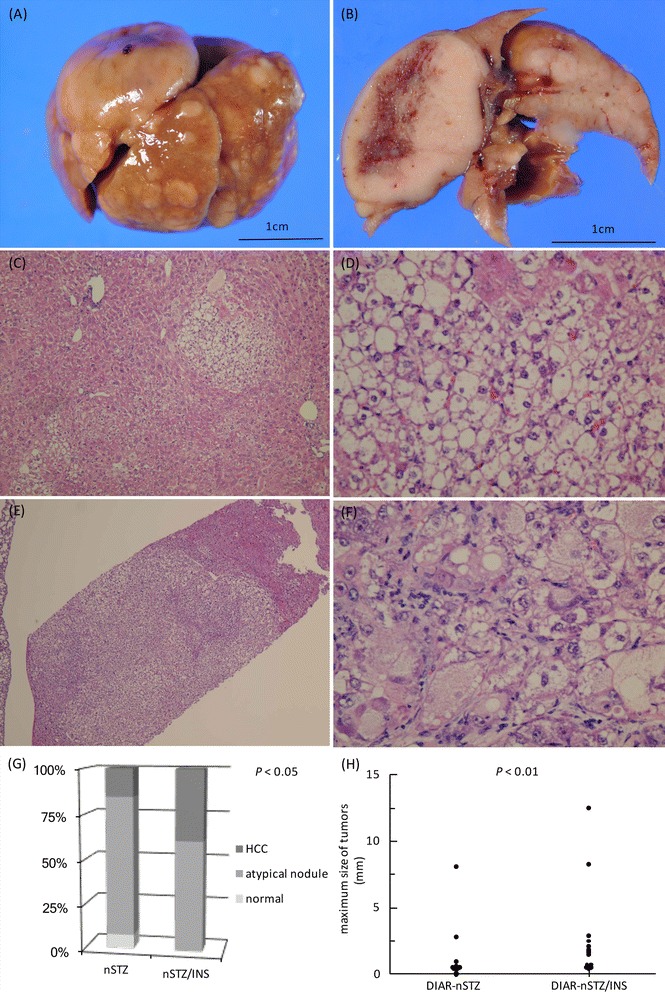
Histopathological findings in mice livers after treatment. **A**, **B** Macroscopic image of the liver of DIAR-nSTZ/INS mice. **C**, **D** Dysplastic nodules observed in DIAR-nSTZ mice [hematoxylin and eosin staining; **C** ×40, **D** ×100]. **E**, **F** HCC observed in DIAR-nSTZ/INS mice [hematoxylin and eosin staining; **E** ×40, **F** ×100]. **G** Comparison of pathological findings. **H** Comparison of maximum tumor size

Multiple tumors were macroscopically observed in the livers of both DIAR-nSTZ and DIAR-nSTZ/INS mice (Fig. 3A, B). Histologically, in the livers of the DIAR-nSTZ mice, HCC was observed in 15% of cases and dysplastic nodules (DNs) were observed in 77% of cases (Fig. 3C, D). In the livers of DIAR-nSTZ/INS mice, HCC was observed in 39% of cases and DNs were observed in 61% of cases (Fig. 3E, F). The HCC occurrence rate at 12 weeks of age was significantly higher in the DIAR-nSTZ/INS mice compared to the DIAR-nSTZ mice (Fig. 3G). Moreover, the average tumor size was significantly larger in the STZ/INS-treated mice than in the STZ-treated mice (Fig. 3H).

All mice in the control and the STZ-treated groups survived during observation. On the other hand, the survival rate of the DIAR nSTZ/INS mice at 12 weeks of age was 75%, and tended to be lower than that in the other groups (*p* = 0.51). In the livers of three mice that died after 10 weeks of age, large HCCs were observed.

**Fig. 4:**
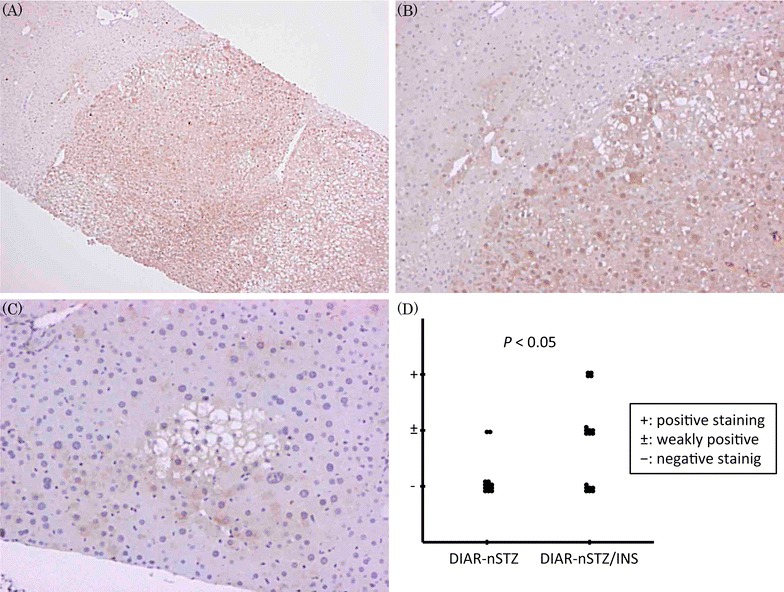
Immunohistochemical analysis of ERK1/2 expression. **A**, **B** Tumors in DIAR-nSTZ/INS mice [**A** ×40, **B** ×100]. **C** Tumors in DIAR-nSTZ mice [**C** ×100]. **D** Immunohistochemistry scoring for ERK1/2

In 11/18 DIAR nSTZ/INS mice, nuclear expression of ERK1/2 was observed in the tumor area. In contrast, nuclear expression was not observed in the non-tumor area of either the DIAR nSTZ or the nSTZ/INS mice. Although nuclear expression of ERK1/2 was observed in a few DIAR nSTZ mice (Fig. 4C), it was significantly specific to the DIAR nSTZ/INS mice (Fig. 4D). The expression of ERK1/2 was more frequently observed in the livers with dysplastic nodules than in the livers with HCC.

## Discussion

The present study has provided two important findings. Insulin treatment improved hyperglycemia, secondary weight loss, and dyslipidemia caused by hyperglycemia in DIAR-nSTZ mice. However, insulin treatment promoted, rather than inhibited, the progression of liver carcinogenesis. Our results suggest that hyperinsulinemia rather than hyperglycemia can accelerate the progression of HCC. Furthermore, immunohistochemical analysis of ERK1/2 suggests that activation of insulin signaling may accelerate tumor progression in the hyperglycemic state.

Several studies have reported that insulin treatment is a risk factor for HCC [16–18], although other studies have shown that insulin use is not associated with the incidence of malignancy [19, 20]. In the clinical setting, insulin treatment for HCC patients with DM is not uncommon. It is still unclear whether insulin treatment worsens the prognosis of HCC patients because poor blood glucose control also worsens their prognosis. The aim of the present study was to investigate in vivo, whether the benefit of insulin treatment overrides its tumor proliferating effect.

The mechanisms underlying the association between exogenous insulin and HCC are unclear but there are several possibilities. First, insulin is an important mitogen and stimulates cell proliferation [21]. Insulin directly upregulates intracellular molecules such as mitogen-activated protein kinase, involved in cell proliferation, by binding to insulin receptors [22]. Indeed, the current study demonstrated nuclear expression of ERK1/2 was significantly higher in DIAR-nSTZ mice that received insulin treatment compared to mice without insulin treatment. In addition, suppressors of intracellular insulin signaling, such as tensin homology deleted on chromosome 10 and the SH2 domain containing inositol phosphatase-2, are downregulated in HCC; therefore, the effects of insulin could be enhanced in HCC [23, 24]. The expression of these suppressors was not examined in the current study. However, our result showing higher expression of ERK1/2 in the tumor area compared to the non-tumor area is compatible with this hypothesis. Moreover, insulin interacts with the insulin-like growth factor (IGF)-1 receptor [25], resulting in the activation of tyrosine kinase and a cascade of intracellular responses. Insulin also inhibits the binding of IGF-1 to IGF-binding proteins, causing an increase in IGF-1 levels. The IGF system is a potent growth regulator closely associated with carcinogenesis [26]. Indeed, serum IGF-I level is reported to be an independent predictor of recurrence and survival in early HCC [27]. Although we did not evaluate IGF-1 serum levels, ERK1/2 are known to be downstream substrates of IGF-1 signaling as well as insulin signaling.

The present study has several limitations. First, we did not assess the effect of hyperglycemia on tumor progression. This would require evaluating the effects of blood glucose control by antidiabetic medications other than insulin on the incidence and/or severity of HCC in DIAR-nSTZ mice. The use of metformin is reported to be associated with a decreased risk of cancer [28] and a meta-analysis of observational studies showed a 50% reduction in HCC incidence [17]. Second, DIAR-nSTZ is a chemically induced model of HCC and so we focused on the promotion and/or progression but not the initiation action of hyperinsulinemia. Although insulin is thought to work as a tumor promoter or progressor via its effect on cell proliferation, additional studies in other animal models are needed to examine the potential of insulin as a tumor initiator. In addition, we did not examine the relationship between insulin dose and the facilitatory effect on HCC development. Additional experiments with varying doses of insulin are needed to examine the dose-dependent effect.

## Conclusions

Insulin treatment promoted, rather than inhibited, the progression of liver carcinogenesis in DIAR-nSTZ mice. Hyperinsulinemia rather than hyperglycemia can accelerate the progression of HCC via insulin signaling. Therefore, an alternative to insulin treatment for HCC patients with DM should be considered, if possible. Further studies are necessary to evaluate the carcinogenic effect of hyperglycemia itself.

## Abbreviations

HCC: hepatocellular carcinoma
STZ: streptozotocin
DIAR: ddY, Institute for Animal Reproduction
DM: diabetes mellitus
DN: dysplastic nodules
IGF: insulin-like growth factor
